# How healthy and processed are foods and drinks promoted in supermarket sales flyers? A cross-sectional study in the Netherlands

**DOI:** 10.1017/S1368980021001233

**Published:** 2021-04-12

**Authors:** Audrey Hendriksen, Romy Jansen, Sanne Coosje Dijkstra, Marlijn Huitink, Jacob C Seidell, Maartje P Poelman

**Affiliations:** 1 Department of Health Sciences, Faculty of Science, Vrije Universiteit Amsterdam, Amsterdam Public Health Research Institute, Amsterdam, The Netherlands; 2 Chair group Consumption and Healthy Lifestyles, Wageningen University and Research, 8130, 6700 EW Wageningen, The Netherlands

**Keywords:** Supermarkets, Sales flyers, Food environment, Price promotions, Ultra-processed food

## Abstract

**Objective:**

To investigate to what extent promotions in Dutch supermarket sales flyers contribute to a healthy diet and whether there are differences between supermarket types.

**Design:**

A cross-sectional study investigating promotions on foods and beverages (*n* 7825) in supermarket sales flyers from thirteen Dutch supermarket chains (8-week period), including ten traditional, two discount and one organic supermarket chain(s). Promoted products were categorised by food group (e.g. bread), contribution to a healthy diet (yes/no), degree of processing (e.g. ultra-processed), promotion type (temporary reduction in price, volume-based promotions or advertised only) and percentage discount of price promotions. Differences between supermarket chains in the degree of healthiness and processing of products and the types of price promotions were investigated.

**Results:**

In total, 70·7 % of all promoted products in supermarket sales flyers did not contribute to a healthy diet and 56·6 % was ultra-processed. The average discount on less healthy products (28·7 %) was similar to that of healthy products (28·9 %). Less healthy products were more frequently promoted via volume-based promotions than healthy products (37·6 % *v*. 25·4 %, *P* < 0·001). Discount supermarket chains promoted less healthy (80·3 %) and ultra-processed (65·1 %) products more often than traditional supermarket chains (69·6 % and 56·6 %, respectively).

**Conclusions:**

The majority of promoted products via supermarket sales flyers do not contribute to a healthy diet. As promotions are an important determinant of food purchasing decisions, supermarkets do not support healthy choices. Future studies should identify barriers that withhold supermarket chains from promoting more healthy foods in supermarket sales flyers.

Unhealthy diets are an important risk factor for the development of overweight, obesity and other nutrition-related non-communicable diseases and are therefore a global public health threat^(1–3)^. A high intake of saturated fats, sugar and salt combined with a low intake of fish, fibre, vegetables and fruit characterises unhealthy dietary patterns, which are common in the Dutch population^(4,5)^. As a consequence, about half of the adult Dutch population is overweight and roughly 15 % has obesity^(6)^. Despite continuous efforts to promote healthy diets, the consumption of energy-dense and nutrient-poor foods and drinks has increased in many high-income countries over the past decades, including the Netherlands^(7–9)^.

The retail food environment, which has been defined as the availability and accessibility of foods in people’s daily environment, has increasingly been recognised as a dominant determinant of food choice^(10–13)^. Within this retail food environment, supermarkets have become a major source for individual and household food purchases^(14–17)^. This is also the case in the Netherlands, where the majority of household food budget is spent in supermarkets^(5)^. Supermarkets use different marketing techniques to encourage food purchases^(18–20)^. Among the main and most effective marketing techniques are the use of price promotions. Supermarket sales flyers are an important way of informing consumers about their assortment and discounts^(13,21,22)^. In this way, such supermarket sales flyers can influence food purchasing decisions^(23)^, including product advertisement (no discount), temporary reduction in price (e.g. 10 % discount) or volume-based promotions (multi-buy), which are effective in increasing sales of the promoted products^(24–28)^.

Previous studies investigating the content of supermarket sales flyers found that most promoted products did not contribute to a healthy diet^(12,15,18–20,24)^. A recent study collected weekly online price data for 1 year from the largest Australian supermarket chain and found that price promotions were more prevalent and greater in magnitude for unhealthy foods than for healthy foods^(29)^. Additionally, studies conducted in the UK found that customers that buy on promotions intend to buy greater amounts of less healthy products and less fruit and vegetables^(30,31)^. To our knowledge, literature on the promotions in supermarkets in the Netherlands is sparse. In a study from the Netherlands published in 2015, it was found that the majority of food promotions in supermarket sales flyers of four leading supermarket chains over an 8-week period were predominantly unhealthy (66·7 %)^(24)^. They also reported significant differences in the number of healthy promotions (33·3 % *v*. 19·0 %) and the percentage discount of promoted products (28·0 % *v*. 21·0 %) between traditional and discount supermarkets. However, these findings represented supermarket sales flyers of 2012, and the retail food environment and the promotions of products are likely to have changed since then. For example, recent attention of policy-makers and the media to the importance of the retail food environment and the marketing of foods and drinks for public health has encouraged retailers to express their intentions to increase their efforts to support healthy food choices. As part of the Dutch National Prevention Agreement, supermarkets agreed to create a healthier food environment by promoting the purchase of foods and drinks that are compatible with the Dutch guidelines for a healthy diet^(32)^. However, the implementation of these intentions is often vaguely described, and it is largely unclear which concrete actions are carried out to support a healthy diet. Besides, the degree of food processing of promoted products, which has become of interest due to a shift in food consumption from basic foods to (ultra-)processed foods in many countries, including the Netherlands^(8,33)^, has not been investigated for promotions in supermarket sales flyers. This is of interest because ultra-processed foods are often cheap, energy-dense and nutrient-poor^(25)^ and have been associated with increased energetic intake and weight gain^(26)^.

Therefore, the overall aim of this study was to investigate to what extent products promoted in Dutch supermarket sales flyers contribute to a healthy diet and whether there are differences between supermarket types. The results of this study may provide insights for the direction of policy and interventions to improve the healthiness of retail food environments, which in turn is likely to improve dietary behaviours.

## Methods

### Study design

A cross-sectional study was conducted, examining the products promoted via weekly online supermarket sales flyers of thirteen supermarket chains in the Netherlands, issued between February and April 2018.

### Procedure and data collection

To extract the products promoted in supermarket sales flyers, we aimed to include most supermarket chains in the Netherlands based on degree of market share^(34–36)^. In total, thirty-one unique supermarket chains were identified and characteristics were recorded (e.g. market share and type of supermarket). Only supermarket chains that distributed a weekly online sales flyer, which was identical in every region in the Netherlands, were included. Nine supermarket chains were excluded as a result of this criteria, leaving twenty-two supermarkets chains. Of these, a sample of ten traditional, two discount and one organic supermarket chains were selected, representing 95 % of national market share of all supermarket chains in 2018 in the Netherlands (Appendix 1). As our intention was to study the healthiness of food promotions in Dutch supermarket sales flyers, we avoid singling out particular supermarket chains and names are deliberately not declared.

All supermarket sales flyers (*n* 103) were sourced weekly online from the supermarket websites. To ensure reliability and accuracy, two researchers (AH and RJ) performed the data extraction of all supermarket sales flyers independently. Discrepancies were resolved and reviewed by the research team until unanimous agreement was achieved.

For the data extraction of the supermarket sales flyers, a pre-defined checklist was developed and used to collect the data. All products promoted in sales flyers were recorded and coded for supermarket name, type of supermarket chain (traditional, discount and organic) and were pooled over the 8-week period. If products from different product categories were promoted in one promotion (e.g. oranges (fruit) and yoghurt (milk and milk products), they were counted as two different product promotions. If similar products were promoted in one promotion (e.g. oranges and apples), they were calculated as one product promotion (fruit). Therefore, the number of promoted products included in the analyses exceeds the number of actual promotions that are analysed. Promoted non-food products were not recorded. Also, promotions including all products from a particular brand that represented a range of different food groups (e.g. *Go-Tan* sweet sauces and *Go-Tan* whole wheat noodles) were placed in a separate group ‘products not able to extract’ and excluded from data analysis (*n* 20 promotions).

### Measures

Products promoted were first categorised per food group. Subsequently, products promoted were categorised with respect to their contribution to a healthy diet (yes/no), degree of processing, usual product price, promotional product price and type of promotion.

#### Food groups

All recorded products promoted via supermarket sales flyers were classified in food groups based on the description and categorisation of the Dutch Food Composition Database (also called the ‘NEVO table’)^(37)^. The NEVO table contains data on the composition of foods and drinks consumed frequently by a large part of the Dutch population. For the purpose of this study, separate food groups were created for ‘Alcoholic drinks’ and ‘Non-alcoholic drinks’, since the NEVO table categorises both into the same food group. Additionally, unprepared tea and coffee in beans, powder, pad or cup form were placed in a separate food group, ‘Unprepared tea and coffee’, instead of the original NEVO food group ‘Alcoholic and non-alcoholic beverages’ as having incomparable nutritional values to liquid beverages. Lastly, olives were placed in the food group ‘Nuts, seeds and snacks’ instead of ‘Fruit’ (where olives were originally categorised according to the NEVO table), because olives are more typically consumed as a snack.

Some advertised products were not explicitly described in the NEVO table. These products were compared with similar products and placed in their most corresponding food group. For example, cheese straws of puff pastry were not included in the NEVO table and placed in the same food group as cheese rolls of puff pastry (‘Nuts, seeds and snacks’). Appendix 2 provides details about how the promoted products were classified over the twenty-five food groups.

#### Contribution to a healthy diet (‘healthiness’) of promoted products

To assess the contribution to a healthy diet (yes/no), all promoted products were assessed according to the *Wheel of Five* of the Netherlands Nutrition Centre that identifies all products that are included in the Dutch guidelines for a healthy diet^(38,39)^. For the purpose of this study, promoted products were categorised as ‘healthy’ or ‘less healthy’, using the ‘Do I choose healthy?’ app that integrates the criteria of the Wheel of Five. If a specific product was not available in the app, the nutrition facts label derived from the supermarket website was examined and manually classified by the researchers based on criteria of the Wheel of Five^(38,39)^. If the supermarket website did not provide the warranted product information to manually classify products, the nutrition facts label derived in the supermarket store was examined. In some cases, these products were not available in the supermarket and the nutrition facts label of a similar product offered by the same supermarket chain was used as reference instead (exact numbers were not recorded, we estimate that this was the case at less than ten products). If both healthy and less healthy foods and drinks were promoted together in one promotion, this promotion was classified in a separate category (‘varying healthiness’).

#### Degree of processing of products promoted

All products were categorised in either ‘Unprocessed/minimally processed foods’, ‘Processed culinary ingredients’, ‘Processed foods’ or ‘Ultra-processed food and drink products’, based on the NOVA food processing classification(40). We were able to classify most products in their category based on the correspondence of the product’s nutrition facts label and the criteria of the NOVA food processing classification. In case the nutrition facts label was not available, the same procedure was followed as in the categorisation in Wheel of Five categories. If products of different food processing categories were promoted together in one promotion, this promotion was classified in a separate category (‘Varying in degree of food processing’). Appendix 3 shows the four NOVA food processing categories with explanation and examples of products per category.

#### Percentage discount on promoted products

Based on the usual product price and the promotional product price, percentage discount was calculated for every single promotion. If a promotion consisted of multiple products with varying prices, the average price across the price range, and subsequently the average percentage discount, was calculated.

#### Type of product promotions

For all products in supermarket sales flyers, the type of promotion was determined. In total thirty-six types of promotions were observed (outlined in Appendix 4), which were combined into three promotion groups: temporary reduction in price (e.g. 10 % discount), volume-based promotions (multi-buy discount) and advertised only (no discount).

### Data analysis

In line with a similar Dutch study^(24)^, all promoted products in a given week were pooled for the analyses. In total, there were promotions for 7825 products in the supermarket sales flyers over an 8-week period and these were the unit of analysis. Descriptive analyses were performed to gain insight into the healthiness of the promoted products, the distribution of the promoted products across the different food groups and the percentage discount of the price promotions per food group. Those healthy/less healthy products that were most often promoted and those products with the highest discount per food group and per supermarket type were examined.

Secondly, descriptive analyses and one-way ANOVA analyses were performed to study whether there were differences in percentage discount of the promoted products per healthiness category. ANOVA analyses were also performed to investigate differences between supermarket types in percentage discount of the promoted products per healthiness category and the degree of processing of the promoted products. In the statistical analyses, the products which were advertised only (no discount) were excluded when examining the percentage discount between promoted products per healthiness category or different types of supermarkets (*n* 1623 (20·7 %)).

Thirdly, to investigate whether there was a difference in types of promotions for the discounted food products per healthiness category, degree of processing and per supermarket type, chi-square analyses (Bonferroni adjusted) were performed. Furthermore, to investigate differences in the frequency of promoted products per healthiness category and degree of processing between supermarket types (traditional *v*. discount and traditional *v*. organic), chi-square analyses were performed. Two-sided *P*-values of <0·05 were considered statistically significant. All analyses were conducted using the IBM SPSS statistical software package, version 25.0.

## Results

Table 1 provides an overview of the 7825 promoted products, specified by food group, and shows the healthiness, degree of processing, type of promotion and average discount per food group.

**Table 1:** Frequency of the healthiness, degree of processing, average discount and type of promotion of products (*n* 7825) promoted in supermarket chain sales flyers over an eight week period, stratified per food group

Food groups			Healthiness of the promoted products*				Degree of processing of the promoted products†								Type of promotion					
	Total promoted products		Healthy promoted products		Less healthy promoted products		Unprocessed/minimally processed		Processed		Ultra-processed		Average discount‡		Temporary reduction in price		Volume-based price promotion		Advertised only (No discount)	
	*n*	%	*n*	%	*n*	%	*n*	%	*n*	%	*n*	%	Mean %	sd	*n*	%	*n*	%	*n*	%
Total	7825	100	5536	70·7	1669	21·3	1595	20·4	1135	14·5	4426	56·6	29·0	10·6	3965	50·7	2237	28·6	1623	20·7
Vegetables	506	6·5	455	89·9	31	6·1	455	89·9	17	3·4	23	2·6	30·5	10·0	276	54·5	127	25·1	103	20·4
Fruit	406	5·2	370	91·1	10	2·5	372	91·6	14	3·4	2	0·5	30·4	9·9	242	60·0	82	20·2	82	20·2
Bread	542	6·9	22	4·1	423	78·0	1	0·2	70	12·9	453	83·6	28·7	10·9	228	42·1	165	30·4	149	27·5
Cereal and cereal products	161	2·1	8	5·0	94	58·4	71	44·1	17	10·6	46	28·6	30·9	12·5	63	39·1	76	47·2	22	13·7
Potatoes	164	2·1	94	57·3	55	33·5	85	51·8	25	15·2	36	22·0	31·5	11·9	81	49·4	44	26·8	39	23·8
Fish	253	3·2	192	75·9	31	12·3	55	21·7	78	30·8	76	30·0	26·9	8·2	143	56·5	30	11·9	80	31·6
Legumes	33	0·4	3	9·1	1	3·0	3	9·1	4	12·1	–	–	36·4	10·3	8	24·2	24	72·7	1	3·0
Meat, meat products and poultry	1011	12·9	183	18·1	764	75·6	20	20·1	64	6·3	638	63·1	26·2	9·8	619	61·2	135	13·4	257	25·4
Soy products and vegetarian products	103	1·3	6	5·8	69	67·0	32	1·9	12	11·7	58	56·3	30·1	10·0	52	50·5	24	23·3	27	26·2
Eggs	19	0·2	19	100	–	–	19	100	–	–	–	–	21·3	8·4	8	42·1	–	–	11	57·9
Nuts, seeds and snacks	405	5·2	19	4·7	360	88·9	16	4·0	27	6·7	333	82·2	29·0	10·5	159	39·3	175	43·2	71	17·5
Milk and milk products	390	5·0	19	4·9	267	68·5	30	7·7	11	2·8	240	61·5	29·2	10·1	181	46·4	148	37·9	61	15·6
Cheese	355	4·5	28	7·9	263	74·1	–	–	114	32·1	216	60·8	28·0	9·3	202	59·9	69	19·4	84	23·7
Alcoholic beverages	871	11·1	–	–	871	100	–	–	591	67·9	273	31·3	27·9	10·1	631	72·4	148	17·0	92	10·6
Non-alcoholic beverages	442	5·6	23	5·2	405	91·6	81	18·3	19	4·3	270	61·1	31·5	11·1	197	44·6	215	48·6	30	6·8
Unprepared tea and coffee	185	2·4	165	89·2	7	3·8	165	89·2	1	0·5	6	3·2	30·8	9·5	90	48·6	88	47·6	7	3·8
Fats, oils and savoury sauces	274	3·5	35	12·8	226	82·5	1	0·4	33	12·0	186	67·9	30·7	11·2	115	42·0	106	38·7	53	19·3
Soups	96	1·2	1	1·0	95	99·0	–	–	8	8·3	88	91·7	34·6	11·5	25	26·0	57	59·4	14	14·6
Mixed dishes	371	4·7	–	–	371	100	1	0·3	3	0·8	363	97·8	31·1	11·2	157	42·3	126	34·0	88	23·7
Savoury bread spreads	89	1·1	2	2·2	87	97·8	2	2·2	16	18·0	70	78·7	32·6	8·2	41	46·1	43	48·3	5	5·6
Sugar, sweets and sweet sauces	469	6·0	–	–	468	99·8	1	0·2	10	2·1	447	95·3	24·2	10·5	179	38·2	129	27·5	161	34·3
Pastry and biscuits	556	7·1	–	–	551	99·1	–	–	6	1·1	542	97·5	28·4	10·6	230	41·4	164	29·5	162	29·1
Herbs and spices	116	1·5	25	21·6	79	68·1	29	25·0	7	6·0	69	59·5	31·2	11·1	34	29·3	62	53·4	20	17·2
Clinical formulas	2	0·03	–	–	2	100	1	50·0	–	–	1	50	20	§	1	50	–	–	1	50
Miscellaneous foods	6	0·1	–	–	6	100	2	33·3	3	50·0	–	–	17·6	5·2	3	50	–	–	3	50

* Varying healthiness category (*n* 620 products) are not presented.

† ‘Processed Culinary Ingredients’ (*n* 41) and ‘Varying in degree of food processing’ (*n* 628) categories are not presented.

‡ Promoted products without discount (0 %) were excluded from data analysis (*n* 1623).

§ No SD because discount presented is for *n* 1.

### Healthiness of promoted products (per food group and supermarket type)

In total, 21·3 % of all promoted products were healthy, whereas 70·7 % of the promoted products were less healthy. The top five most promoted products per food group were categorised as ‘Meat, meat products and poultry’ (12·9 %), followed by ‘Alcoholic beverages’ (11·1 %), ‘Pastry and biscuits’ (7·1 %), ‘Bread’ (6·9 %) and ‘Vegetables’ (6·5 %). In comparison with traditional supermarket chains, discount supermarket chains promoted a higher proportion of less healthy products (80·3 % *v*. 69·6 %, *χ* ^2^ = 53·40, *P* =< 0·001) and a lower proportion of healthy products (18·6 % *v*. 21·2 %, *χ* ^2^ = 3·99, *P* = 0·046). Organic supermarket chains promoted a lower proportion of less healthy products (56·2 % *v*. 69·6 %, *χ* ^2^ = 18·41, *P* =< 0·001) and a higher proportion of healthy products (37·6 % *v*. 21·2 %, *χ* ^2^ = 34·39, *P* =< 0·001), compared to traditional supermarket chains (Table 2).

**Table 2:** Frequency of the healthiness, degree of processing, average discount and type of promotion of products (*n* 7825) promoted in supermarket chain sales flyers over an eight week period, stratified by type of supermarket (traditional, discount, organic chains)

			Healthiness of the promoted products*				Degree of processing of the promoted products†								Type of promotion‡			
	Total promoted products		Healthy promoted products‡		Less healthy promoted products‡		Unprocessed/minimally processed		Processed		Ultra-processed		Average discount‡		Temporary reduction in price†		Volume-based promotions†	
	*n*	%	*n*	%	*n*	%	*n*	%	*n*	%	*n*	%	M	sd	*n*	%	*n*	%
Total	7825	100	1669	21·3	5536	70·7	1595	20·4	1135	14·5	4426	56·6	29·0	10·6	3965	63·9	2237	36·1
Traditional	6481	82·8	1376	21·2	4511	69·6	1374	20·4	870	13·4	3668	56·6	29·3	10·7	3549	62·0	2174	38·0
Discount	1118	14·3	208	18·6**	898	80·3***	178	15·9****	183	16·4	728	65·1****	26·3	7·2****	195	75·9****	62	24·1****
Organic	226	2·9	85	37·6***	127	56·2***	93	41·2****	82	36·3****	30	13·3****	23·5	8·3****	221	99·5****	1	0·5****

Significant difference *v*. traditional supermarket: **P < 0·05, ***P < 0·01, ****P < 0·001.

* Varying healthiness category (*n* 620 products) are not presented.

† Data of ‘Processed Culinary Ingredients’ (*n* 41) and ‘Varying in degree of food processing’ (*n* 628) are not presented.

‡ Promoted products without discount (0 %) were excluded from data analysis (*n* 1623).

### Degree of processing of the promoted products (per food group and supermarket type)

The majority of all promoted products were categorised as ‘Ultra-processed’ (56·6 %), followed by 20·4 % as ‘Unprocessed/minimally processed’, 14·5 % as ‘Processed’, 8·0 % as ‘Varying in degree of food processing’ and 0·5 % as ‘Processed culinary ingredients’. The top five of food groups with the highest proportion of promoted products that were categorised as ‘Unprocessed/minimally processed’ were the food groups ‘Eggs’ (100 %), ‘Fruit’ (91·6 %), ‘Vegetables’ (89·9 %), ‘Unprepared tea and coffee’ (89·2 %) and ‘Potatoes’ (51·8 %). The top five of food groups with the highest proportion of promoted products that were categorised as ‘Ultra-processed’ were the groups ‘Mixed dishes’ (97·8 %), ‘Pastry and biscuits’ (97·5 %), ‘Sugar, sweets and sweet sauces’ (95·3 %), ‘Soups’ (91·7 %) and ‘Bread’ (83·6 %), Table 1. Ultra-processed foods were significantly more often promoted by discount supermarket chains (65·1 %) compared to traditional supermarket chains (56·6 %) that in turn promoted more often ultra-processed foods than the organic supermarket chain (13·3 %, *χ* ^2^ = 340, *P* < 0·001) (Table 2).

### Discount on promoted products (per food group, healthiness and degree of processing)

Irrespective of the frequency at which food groups were promoted, the top five products promoted with highest average discount were categorised as ‘Legumes’ (36·4 %), Soups (34·6 %), ‘Savoury bread spreads’ (32·6 %), Non-alcoholic beverages (31·5 %) and Potatoes (31·5 %) (Table 1). In total, the average percentage discount on less healthy products was similar to the average percentage discount on healthy products (28·7 % (sd 10·7) *v*. 28·9 % (sd 10·2), F = 0·20, *P* = 0·65). The average discount on unprocessed/minimally processed (29·4 %, sd = 10·6), processed (29·0 %, sd = 10·2) and ultra-processed foods (28·5 %, sd = 10·7) did not differ statistically.

### Type of promotion per food group, healthiness and degree of processing

Top five products most frequently promoted products via temporary reduction in price per food group were ‘Alcoholic beverages’ (72·4 %), ‘Meat, meat products and poultry’ (61·2 %), ‘Cheese’ (59·9 %), ‘Fruit’ (60·0 %) and ‘Fish’ (56·5 %), Table 1. The food groups ‘Legumes’ (72·7 %), ‘Soups’ (59·4 %), ‘Herbs and spices’ (53·4 %), ‘Non-alcoholic beverages’ (48·6 %) and ‘Savoury bread spreads’ (48·3 %) were most often promoted via volume-based price promotions. Healthy promoted products were promoted more often by the use of a temporary reduction in price than less healthy products (74·6 % *v*. 62·4 %, *χ* ^2^ = 82·99, *P* < 0·001). Less healthy products were promoted more often via volume-based price promotions, compared to healthy products (37·6 % *v*. 25·4 %, *χ* ^2^ = 13·25, *P* < 0·001). Ultra-processed foods were also more often promoted via volume-based price promotions than unprocessed/minimally processed products (40·1 % *v*. 27·3 %, *χ* ^2^ = 171·63, *P* < 0·001).

## Discussion

This study showed that the majority of the advertised and discounted products in Dutch supermarket sales flyers did not contribute to a healthy diet (70·7 %) and most were considered to be ultra-processed (56·6 %). Of all food groups, meat and meat products, alcoholic beverages, and pastry and biscuits were most frequently promoted. Moreover, less healthy and ultra-processed products were more frequently promoted through volume-based price promotions compared to healthy and unprocessed/minimally processed products, indicating that consumers who are looking for discounts are often tempted to buy larger quantities of less healthy and ultra-processed foods and drinks. Nevertheless, irrespective of the frequencies of the promoted products, the average magnitude of the discounts between healthy *v*. less healthy and unprocessed/minimally processed *v*. ultra-processed products promoted was not statistically different. We did observe differences between supermarket types. Discount supermarket chains promoted less healthy and ultra-processed products more frequently compared to traditional supermarket chains, while this was opposite for the organic supermarket chain. Traditional supermarket chains did, however, provide the highest average discount, followed by discount supermarkets and organic supermarket chain(s).

Based on our results, it can be concluded that the majority of Dutch supermarket chains do not promote the purchase of healthy products by means of promotions in their sales flyers. This is similar to the observations of Ravensbergen et al showing that 66·7 % of the supermarket circular promotions in 2012 were not in line with national dietary guidelines, despite the growing awareness on the importance of healthy retail environments in the past years^(24)^. In 2018, supermarkets agreed to encourage healthier food choices as part of the National Prevention Agreement^(32)^. Future studies should investigate if this can be achieved by self-regulation by supermarkets or needs to be enforced through government policies. Similar findings have been observed internationally, showing a high ratio of unhealthy-to-healthy products promoted in supermarket circulars in the USA, Australia, the UK, South Africa, Malaysia and Hong Kong^(12,15,18–20,41,42)^. Only supermarket sales flyers from particular supermarket chains in the Philippines, India, Sweden, Singapore and New Zealand seem to promote more healthy than unhealthy products, which was probably due to relatively high proportions of promotions for fruits and vegetables^(20)^. A possible explanation for the results of our study might be that it is more attractive for supermarkets to promote easy to store products with a longer shelf life, representing mainly less healthy and ultra-processed products, due to the fact that healthy fresh products are often difficult to stock and are less successful in boosting sales^(43)^.

An alarming finding of our study was that the frequency of promoted alcoholic drinks in our study has almost doubled compared to results of 2012 (11·1 % *v*. 6·3 %), although it should be considered that our study included a higher number of supermarket chains and therefore results are not fully comparable. In concordance with our results, studies from Australia, New Zealand and the UK showed a high percentage of alcoholic drinks (>12 %) in their circular promotions^(20)^.

Our results also support prior findings indicating that discount supermarket chains offer the least supportive environment for healthy food choices/purchases, compared to traditional and the organic supermarket chain^(24,44,45)^. Discount supermarket chains mainly attract consumers with a lower income, who have a higher sensitivity to lower prices compared to consumers with a higher socio-economic status^(8,14)^ and are also suffering more often from diet-related non-communicable diseases. On the contrary, the organic supermarket chain seem to offer the most supportive environment for healthy food purchases, promoting the highest proportion of healthy and unprocessed/minimally processed products compared to the other supermarket types (despite still half of the circular content of the organic supermarket chain included less healthy products). Average price discounts on products of the organic supermarket chain were lower than that of traditional supermarket chains. Notwithstanding the results that the organic supermarket had the healthiest flyers, it is well known that products sold are more expensive for consumers than discount and traditional supermarkets and thus less attractive for consumers with a low income.

This study has several strengths. First, data were collected for a variety of 13 supermarket chains, representing 95 % of total national market share and a broad range of consumer profiles and supermarket types. This increases the generalisability of the results, at least within the Netherlands. Second, promotions that represented two or more different food groups were recorded separately for each food group. Therefore, it was possible to limit the number of promoted products that could not be categorised and to assess supermarket sales flyers at the level of products per promoted food group. Third, this study is one of the first to explore the degree of food processing of promoted products in supermarket sales flyers. Ultra-processing of foods and drinks has become a more important determinant for the development of nutrition-related diseases and results of this study could be used for comparison with future international research^(43)^. Lastly, all types of promoted foods and drinks in all supermarket sales flyers were taken into account, which gives a complete overview of the range of products. Some studies only examined the first page of each circular, which could lead to an overestimation of food groups often advertised on the first page^(12,21,44,45)^. However, limitations of this study must be noted as well. In accordance with other studies and to compare our results, this study was limited to an 8-week time frame for data collection and it is unclear how the results are generalisable across the year. We were also not able to test for seasonal variations in product promotions, and products may have been promoted more or less frequently due to seasonal dependency^(29)^. In addition, the collection period included Valentine’s Day and Easter and, as a consequence, some particular products that are typically associated with festive occasions may have been promoted more frequently than usual (e.g. pastries and chocolates). However, a period of 8 weeks seems to be sufficient to reduce the effects of weekly variations in promoted products^(20)^. Furthermore, the current study focused on the content of supermarket sales flyers, even though other food environmental exposures in supermarkets, such as in-store location and discount information in other media, as well as less obvious marketing techniques, such as shape and size of portions and brand association, could also influence the purchase behaviour of consumers^(16,17,46)^. At last, this study included only two discount and one organic supermarket chain(s) compared to ten traditional supermarket chains. Therefore, findings between supermarket types may have been influenced as a result of the large variation in sample sizes. Especially results concerning the differences between the organic supermarket chains and traditional supermarket chains should be interpreted with caution, due to the small number of promoted products for the organic supermarket chain.

Since supermarkets have a major and increasing influence on food purchases, it would be promising if further research investigates the effects of a healthier content of supermarket sales flyers on the actual purchasing and consumption behaviours. It is not only important for supermarkets to discount healthier foods more often, it is also of is also particular importantance that unhealthy foods are less frequently promoted. The relations between food pricing and purchases, particularly in low-income households, are complex^(47)^. Future research should focus on the broader food system and investigate leverage points within the food system that can influence product and price promotions. Moreover, policy-makers can contribute to the creation of a healthier (retail) food environment by implementing policies with regard to the regulation of price promotions on healthy and unhealthy foods in supermarket sales flyers.

## Conclusion

This study demonstrated that Dutch supermarket chains predominantly implement promotions to products that are not recommended in the national dietary guidelines. This may contribute to purchases and consumption of unhealthy products which, in turn, may contribute to the development of nutrition-related chronic diseases. Since Dutch supermarkets have committed themselves to encourage consumers to eat a healthy diet, a suitable way would be to increase the ratio of healthy-to-unhealthy promoted products in order to facilitate purchases of healthier products. More research is needed to determine whether these changes will contribute to healthier food purchases and diets.

## Supplementary material

For supplementary material accompanying this paper visit https://doi.org/10.1017/S1368980021001233. click here to view supplementary material

## References

[1] Lim SS, Vos T, Flaxman AD et al. (2012) A comparative risk assessment of burden of disease and injury attributable to 67 risk factors and risk factor clusters in 21 regions, 1990–2010: a systematic analysis for the Global Burden of Disease Study 2010. Lancet 380, 2224–2260. 23245609 10.1016/S0140-6736(12)61766-8 PMC4156511

[2] World Health Organization (2015) Global Strategy on Diet, Physical Activity and Health: Diet, Nutrition and the Prevention of Chronic Diseases. WHO Tech Rep Series No 916 (TRS 916). Geneva: WHO.

[3] Amine E, Baba N, Belhadj M et al. (2003) Diet, Nutrition and the Prevention of Chronic Diseases. In Report of a Joint WHO/FAO Expert Consultation. Geneva: World Health Organisation.

[4] Bamia C, Trichopoulos D, Ferrari P et al. (2007) Dietary patterns and survival of older Europeans: the EPIC-Elderly Study (European Prospective Investigation into Cancer and Nutrition). Public Health Nutr 10, 590–598. 17381929 10.1017/S1368980007382487

[5] de Vries G, de Hoog J, Stellinga B et al. (2014) Towards a Food Policy (in Dutch: Naar een voedselbeleid). The Netherlands Scientific Council for Government Policy (in Dutch: Wetenschappelijke Raad voor het Regeringsbeleid). The Hague: University Press.

[6] Public Health and Health Care (2016) Overweight, numbers and context (in Dutch: Overgewicht, cijfers en context. https://www.volksgezondheidenzorg.info/onderwerp/overgewicht/cijfers-context/huidige-situatie (accessed February 2018).

[7] Moubarac JC, Parra DC, Cannon G et al. (2014) Food classification systems based on food processing: significance and implications for policies and actions: a systematic literature review and assessment. Curr Obes Rep 3, 256–272. 26626606 10.1007/s13679-014-0092-0

[8] Geurts M, van Bakel AM, van Rossum CTM et al. (2017) Food Consumption in the Netherlands and its Determinants. RIVM Report 2016–0195. Bilthoven: National Institute for Public Health and the Environment (RIVM).

[9] Imamura F, Micha R, Khatibzadeh S et al. (2015) Dietary quality among men and women in 187 countries in 1990 and 2010: a systematic assessment. Lancet Glob Health 3, 132–142. 10.1016/S2214-109X(14)70381-X PMC4342410 25701991

[10] Albert J, Amoroso L, Garnett T et al. (2016) Influencing Food Environments for Healthy Diets. Rome: Food and Agriculture Organizations of the United Nations.

[11] Mattes R & Foster GD (2014) Food environment and obesity. Obesity 22, 2459–2461. 25401929 10.1002/oby.20922

[12] Martin-Biggers J, Yorkin M, Aljallad C et al. (2013) What foods are US supermarkets promoting? A content analysis of supermarket sales circulars. Appetite 62, 160–165. 23228904 10.1016/j.appet.2012.12.001

[13] Glanz K, Sallis JF, Saelens BE et al. (2005) Healthy nutrition environments: concepts and measures. Am J Health Promot 19, 330–333. 15895534 10.4278/0890-1171-19.5.330

[14] Zorbas C, Eyles H, Orellana L et al. (2019) Do purchases of price promoted and generic branded foods and beverages vary according to food category and income level? Evidence from a consumer research panel. Appetite 144, 104481. 31589906 10.1016/j.appet.2019.104481

[15] Jahns L, Scheett AJ, Johnson LK et al. (2016) Diet quality of items advertised in supermarket sales circulars compared to diets of the US Population, as assessed by the Healthy Eating Index-2010. J Acad Nutr Diet 116, 115–122.e111. 26508588 10.1016/j.jand.2015.09.016

[16] Brug J (2008) Determinants of healthy eating: motivation, abilities and environmental opportunities. Fam Pract 25, i50–i55. 18826991 10.1093/fampra/cmn063

[17] Thompson C, Cummins S, Brown T et al. (2013) Understanding interactions with the food environment: an exploration of supermarket food shopping routines in deprived neighbourhoods. Health Place 19, 116–123. 23220374 10.1016/j.healthplace.2012.10.003

[18] Cameron AJ, Sayers SJ, Sacks G et al. (2017) Do the foods advertised in Australian supermarket catalogues reflect national dietary guidelines? Health Promot Int 32, 113–121. 28180259 10.1093/heapro/dav089

[19] Jahns L, Payne CR, Whigham LD et al. (2014) Foods advertised in US weekly supermarket sales circulars over one year: a content analysis. Nutr J 13, 95. 25249348 10.1186/1475-2891-13-95 PMC4182832

[20] Charlton EL, Kähkönen LA, Sacks G et al. (2015) Supermarkets and unhealthy food marketing: an international comparison of the content of supermarket catalogues/circulars. Prev Med 81, 168–173. 26348452 10.1016/j.ypmed.2015.08.023

[21] Hawkes C (2008) Dietary implications of supermarket development: a global perspective. Dev Policy Rev 26, 657–692.

[22] Steenhuis IH, Waterlander WE & de Mul A (2011) Consumer food choices: the role of price and pricing strategies. Public Health Nutr 14, 2220–2226. 21752312 10.1017/S1368980011001637

[23] Geliebter A, Ang IY, Bernales-Korins M et al. (2013) Supermarket discounts of low-energy density foods: effects on purchasing, food intake, and body weight. Obesity 21, E542–E548. 23596089 10.1002/oby.20484

[24] Ravensbergen EA, Waterlander WE, Kroeze W et al. (2015) Healthy or Unhealthy on Sale? A cross-sectional study on the proportion of healthy and unhealthy foods promoted through flyer advertising by supermarkets in the Netherlands. BMC Public Health 15, 470. 25943988 10.1186/s12889-015-1748-8 PMC4492173

[25] Hawkes C (2009) Sales promotions and food consumption. Nutr Rev 67, 333–342. 19519674 10.1111/j.1753-4887.2009.00206.x

[26] Hall KD, Ayuketah A, Brychta R et al. (2020) Ultra-processed diets cause excess calorie intake and weight gain: an inpatient randomized controlled trial of Ad Libitum food intake. M Cell Metab 6, 690. 10.1016/j.cmet.2020.08.014 33027677

[27] Ailawadi KL, Gedenk K, Lutzky C et al. (2007) Decomposition of the sales impact of promotion-induced stockpiling. J Mark Res 44, 450–467.

[28] Familmaleki M, Aghighi A & Hamidi K (2015) Analyzing the influence of sales promotion on customer purchasing behavior. Int J Econ Manag Sci 4, 243.

[29] Riesenberg D, Backholer K, Zorbas C et al. (2019) Price promotions by food category and product healthiness in an Australian Supermarket Chain, 2017–2018. Am J Public Health 109, 1434–1439. 31415196 10.2105/AJPH.2019.305229 PMC6727276

[30] Food Standars Scotland (2016) Monitoring retail purchase and price promotions in Scotland (2010–2016). Foods and drinks purchased into the home in Scotland using data from Kantar WorldPanel. https://www.foodstandards.gov.scot/publications-and-research/publications/monitoring-retail-purchase-and-price-promotions-in-scotland-2010-2016 (accessed February 2018).

[31] Coker T, Rumgay H, Whiteside E et al. (2019) Paying the Price: New Evidence on the Link between Price Promotions, Purchasing of Less Healthy Food and Drink, and Overweight and Obesity in Great Britain. Cancer Research UK. https://euagenda.eu/upload/publications/untitled-209677-ea.pdf (accessed July 2019).

[32] The National Prevention Agreement (2018) Towards a Healthier Netherlands. Den Hague: Ministry of Health Welfare and Sport.

[33] Monteiro CA, Moubarac JC, Cannon G, et al. (2013) Ultra-processed products are becoming dominant in the global food system. Obes Rev 14, 21–28. 24102801 10.1111/obr.12107

[34] Distrifood (2017) Dutch marketshares. http://www.distrifood.nl/service/marktaandelen (accessed February 2018).

[35] Central Food Trade Office Netherlands (2018) De supermarktbranche feiten en cijfers [The supermarket industry facts and figures]. http://www.cbl.nl/de-supermarktbranche/feiten-en-cijfers/ (accessed February 2018).

[36] Nielsen (2017) Marktaandelen supermarkten 2017 [Market shares supermarkets 2017]. http://www.agf.nl/artikel/169858/Nielsen-Marktaandelen-supermarkten-2017 (accessed February 2018).

[37] The Dutch Food Composition Database (NEVO database) (2016) Ministry of Health, Welfare and Sports. https://nevo-online.rivm.nl/ (accessed February 2018).

[38] Brink L, Postma-Smeets A & Stafleu A et al. (2016) Guidelines Wheel of Five (Richtlijnen Schijf van Vijf). Den Haag: Stichting Voedingscentrum Nederland.

[39] Brink E, van Rossum C, Postma-Smeets A et al. (2019) Development of healthy and sustainable food-based dietary guidelines for the Netherlands. Public Health Nutr 22, 2419–2435. 31262374 10.1017/S1368980019001435 PMC7083597

[40] Monteiro C, Cannon G, Levy R et al. (2016) NOVA. The star shines bright. [Food classification. Public Health]. World Nutr 7, 28–38.

[41] Ethan D, Samuel L & Basch CH (2013) An analysis of Bronx-based online grocery store circulars for nutritional content of food and beverage products. J Community Health 38, 521–528. 23203239 10.1007/s10900-012-9643-z

[42] Ethan D, Basch CH, Rajan S et al. (2014) A comparison of the nutritional quality of food products advertised in grocery store circulars of high- versus low-income New York City zip codes. Int J Environ Res Public Health 11, 537–547. 10.3390/ijerph110100537 PMC3924459 24384775

[43] Monteiro CA, Cannon G, Moubarac JC et al. (2018) The UN Decade of Nutrition, the NOVA food classification and the trouble with ultra-processing. Public Health Nutr 21, 5–17. 28322183 10.1017/S1368980017000234 PMC10261019

[44] Black C, Ntani G, Inskip H et al. (2014) Measuring the healthfulness of food retail stores: variations by store type and neighbourhood deprivation. Int J Behav Nutr Phys Act 11, 69. 24884529 10.1186/1479-5868-11-69 PMC4132210

[45] Camden A, Levy J, Bassil K et al. (2018) A census of midsize to large supermarkets in toronto: a cross-sectional analysis of the consumer nutrition environment. J Nutr Educ Behav 50, 573–581. 29496398 10.1016/j.jneb.2017.12.002

[46] Chandon P & Wansink B (2012) Does food marketing need to make us fat? A review and solutions. Nutr Rev 70, 571–593. 23035805 10.1111/j.1753-4887.2012.00518.x PMC3495296

[47] Bennett R, Zorbas C, Huse O et al. (2020) Prevalence of healthy and unhealthy food and beverage price promotions and their potential influence on shopper purchasing behaviour: a systematic review of the literature. Obes Rev 21, e12948. 31633289 10.1111/obr.12948

