# Supplementary material for: How healthy and processed are foods and drinks promoted in supermarket sales flyers? A cross-sectional study in the Netherlands
Source: Public Health Nutr. 2021 Apr 12;24(10):3000–8. doi: 10.1017/S1368980021001233 (PMC9884785; doi:10.1017/S1368980021001233)
Supplement: Supplementary file 1 [file S1368980021001233sup001.docx]

**Appendix 1. Characteristics of the included supermarkets and their sales flyers**

| Supermarket chain | Market share in the Netherlands‡ | Supermarket type | Number of stores throughout the Netherlands‡ | Number of circulars collected | Total number of foods and drinks | Number of promoted products in sales flyers | |
| --- | --- | --- | --- | --- | --- | --- | --- |
|  | % |  | N | N | N | Mean | SD |
| 1 | 35.3 | Traditional | 962 | 8 | 524 | 66.3 | 14.7 |
| 2 | 18.7 | Traditional | 583 | 8 | 232 | 29.0 | 3.8 |
| 3 | 10.5 | Discounter | 412 | 8 | 707 | 88.4 | 25.9 |
| 4 | 6.7 | Discounter | 490 | 8 | 411 | 51.4 | 8.0 |
| 5 | 6.4 | Traditional | 260 | 8 | 612 | 76.6 | 13.4 |
| 6 | 3.8 | Traditional | 120 | 8 | 740 | 92.6 | 30.8 |
| 7 | 3.1 | Traditional | 263 | 8 | 646 | 81.3 | 22.5 |
| 8 | 2.5 | Traditional | 130 | 8 | 651 | 81.6 | 15.3 |
| 9 | 2.2 | Traditional | 82 | 8 | 837 | 104.8 | 45.3 |
| 10 | 2.1 | Traditional | 68 | 8 | 786 | 98.5 | 11.7 |
| 11 | 1.8 | Traditional | 80 | 8 | 744 | 93.3 | 31.9 |
| 12 | 1.6 § | Traditional | 64 § | 8 | 709 | 88.8 | 30.9 |
| 13 | 0.3 \| | Organic | 74 \| | 7 ¶ | 226 | 32.3 | 10.5 |

‡ data for 2017 ^(36)^; § data for 2013 ^(38)^; | data for 2015 ^(37)^; ¶ one circular represented two weeks.

**Appendix 2.** NEVO food group categorization

| **NEVO food groups** | **Wheel of Five categorization** | **Included in the Wheel of Five** | **Not included in the Wheel of Five** |
| --- | --- | --- | --- |
| **1. Vegetables** | 1. Vegetables and fruits | Unprocessed vegetables, canned vegetables without added salt or sugar | Canned vegetables with added salt or sugar |
| **2. Fruits** |  | Unprocessed fresh or frozen fruits and squeezed or dried fruits without added sugar | Coconut, canned fruits in syrup |
| **3. Bread** | 2. Bread, grain or cereal products and potatoes | Whole-grain bread, whole-grain crisp bread with sufficient fibers | White bread, croissant, pancakes or wraps |
| **4. Cereal and cereal products** |  | Oatmeal, whole-wheat flour, whole wheat pasta, brown rice | Grain or cereal products with added salt, white flower, regular pasta, white rice |
| **5. Potatoes** |  | Potatoes or potato products without added salt, prepared without cooking fat or cooking fat included in the Wheel of Five | Potato products with added salt, prepared with cooking fat or cooking fat not included in the Wheel of Five |
| **6. Fish** | 3. Fish, legumes, meat, eggs, nuts and diary | Unprocessed fish | Fish consisting of <70% fish |
| **7. Legumes** |  | Legumes, canned legumes without added sugar and limited salt | Canned legumes with added sugar and salt |
| **8. Meat, meat products and poultry** |  | Unprocessed meat, such as chicken | Processed meat, such as sausages and hamburgers |
| **9. Soy products and vegetarian products** |  | Meat substitutes with limited salt and sufficient iron, vitamin B12/B1 and protein  Vegetarian minced meat, not breaded burger with limited added salt | Meat substitutes with added salt and insufficient iron, vitamin B12/B1 and protein  Falafel, breaded vegetarian burgers |
| **10. Eggs** |  | Egg | - |
| **11. Nuts, seeds and snacks** |  | Nuts without added sugar or salt, nut paste and peanut butter consisting of 100% nuts and without added salt or sugar | Salted or sugared nuts, nut paste and peanut butter with added salt or sugar |
| **12. Milk and milk products** |  | (Semi-) skimmed milk, low- and half fat yoghurt | Full fat milk and yoghurt, pudding, yoghurt drink with added sugar |
| **13. Cheese** |  | 20+, 30+ cheese, mozzarella | 40+, 48+ cheese, blue cheese |
| **14. Alcoholic beverages** | 4. Drinks | - | Alcoholic beverages |
| **15. Non-alcoholic beverages** |  | Water, tea and filtered coffee without added sugar or creamer | Coffee and tea with added sugar or creamer, fruit juice, (light) soda |
| **16. Unprepared coffee and tea** |  | Tea bag, coffee powder without added sugar or creamer, coffee beans, pads and cups | Coffee powder with added sugar or creamer |
| **17. Fats, oils and savoury sauces** | 5. Spreading and cooking fats | Plant-based oil, (low-fat) (liquid) margarine | Butter, coconut oil |
| **18. Soups** | Not categorized in Wheel of Five | - | All soups |
| **19. Mixed dishes** |  | - | > 700 kcal  ≤150 grams of vegetables  > 100 grams of meat, fish, eggs, vegetarian products / > 25 grams of nuts / < 60 grams of legumes  ≥ 2 grams of salt per serving  > 15 grams of fats and oils |
| **20. Savoury bread spreads** |  | - | Hummus, sandwich spread |
| **21. Sugar, sweets and sweet sauces** |  | - | Chocolate, liquorice, candy bars, honey |
| **22. Pastry and biscuits** |  | - | All pastry and biscuits |
| **23. Herbs and spices** |  | Cinnamon powder, garlic | Stock powder, herb paste, mustard  Herbs and spices with added salt |
| **24. Clinical formulas** |  | - | All clinical formulas |
| **25. Miscellaneous foods** |  | Vinegar | Coconut milk, (sea) salt |

Adapted from: Brink L, Postma - Smeets A, Stafleu A, Wolvers D. Richtlijnen Schijf van Vijf. Den Haag: Stichting Voedingscentrum Nederland; 2016. (35) & RIVM. NEVO 2016 Food classification (alphabetical). 2016.

Appendix 3. Degree of food processing categorization

According to NOVA, food processing involves “physical, biological and chemical processes that occur after foods separated from nature and before they are consumed or used in the preparation of dishes and meals” ^(40)^. The NOVA food classification categorizes food products according to “the extent and purpose of food processing, rather than in terms of nutrients” and is accepted as a valid tool for varying research areas, including nutrition and public health research ^(7, 40).^

| **Food processing categories and definition** | **Examples** |
| --- | --- |
| **Degree 1 Unprocessed or minimally processed food and drink products** Unprocessed foods are edible parts of plants or of animals and also fungi, algae and water, after separation form nature. Minimally processed foods are natural foods altered by processes such as removal of inedible or unwanted parts, drying, crushing, grinding, fractioning, filtering roasting, boiling, pasteurisation, refrigeration, freezing, placing in containers, vacuum packaging, or non-alcoholic fermentation. None of these processes adds substances such as salt, sugar oils or fats to the original food. | Fresh, squeezed, chilled, frozen, or dried fruits and vegetables; grains including all types of rice; legumes; roots and tubers; fungi; meat, poultry, fish and seafood; eggs; milk; fresh or pasteurised fruit or vegetable juices without added sugar, sweeteners or flavours; pasta, couscous and polenta made with flours, flakes or grits and water; tree and ground nuts and other oil seeds without added salt or sugar; spices and herbs; plain yoghurt with no added sugar or artificial sweeteners added; tea, coffee, drinking water. It also includes foods made up from two or more items in this group; and foods with vitamins and minerals added generally to replace nutrients lost during processing. |
| **Degree 2 Processed culinary ingredients**  Processed culinary ingredients are substances obtained directly from group 1 foods or from nature by processes such as pressing, refining, grinding, milling and spray drying. Group 2 items are rarely consumed in the absence of group 1 foods. | Salt mined or from seawater; sugar and molasses obtained from cane or beet; honey extracted from combs and syrup from maple trees; vegetable oils crushed from olives or seeds; butter and lard obtained from milk and pork; and starches extracted from corn and other plants. Products consisting of two group 2 items remain in this group and they may contain additives used to preserve the product’s original properties. Examples are vegetable oils with added anti-oxidants, cooking salt with added anti-humectants and vinegar with added preservatives that prevent microorganism proliferation. |
| **Degree 3 Processed food and drink products**  Processed foods are relatively simple products made by adding sugar, oil, salt or other group 2 substances to group 1 foods. Most processed foods have two or three ingredients. Processes include various preservation or cooking methods and in the case of breads and cheese, non-alcoholic fermentation. Processed foods may contain additives used to preserve their original properties or to resist microbial contamination. | Canned or bottled vegetables, fruits and legumes; salted or sugared nuts and seeds; salted, cured, or smoked meats; canned fish; fruits in syrup; cheeses and unpackaged freshly made breads. When alcoholic drinks are identified as foods, those produced by fermentation of group 1 foods such as beer, cider and wine, are classified here. |
| **Degree 4 Ultra-processed food and drink products**  Ultra-processed food and drinks are industrial formulations typically with five or more and usually many ingredients. Such ingredients often include those also used in processed foods, such as sugar, oils, fats, salt, anti-oxidants, stabilizers and preservatives. Group 1 foods are a small proportion of or are even absent from ultra-processed products. Classes of additive only found in ultra-processed products include dyes and other colours, colour stabilisers, flavours, flavour enhancers, non-sugar sweeteners, and processing aids such as carbonating, firming, bulking and anti-bulking, de-foaming, anti-caking and glazing agents, emulsifiers, sequestrants and humectants. | Carbonated drinks; packaged snacks; ice-cream, chocolate, candies; mass-produced packaged breads and buns; margarines and spreads; cookies, pastries, cakes, and cake mixes; breakfast cereals, cereal and energy bars; energy drinks; milk drinks, fruit yoghurts and fruit drinks; cocoa drinks; meat and chicken extracts and instant sauces; infant formulas and other baby products; health and slimming products; ready to heat products including pre-prepared pies and pasta and pizza dishes; poultry and fish nuggets and sticks, sausages, burgers, hot dogs, and other reconstituted meat products, and powdered and packaged instant soups, noodles and desserts. When products made solely of group 1 or group 3 foods also contain cosmetic or sensory intensifying additives they are classified here. When alcoholic drinks are produced by fermentation of group 1 foods followed by distillation of the resulting alcohol, such as whisky, gin, rum, vodka, these products are classified here. |

Adapted from: Monteiro C, Cannon G, Levy R, Moubarac J, Jaime P, Martins A, et al. NOVA. The star shines bright. [Food classification. Public Health]. World Nutrition. 2016;7(1-3):28-38) (32)

Appendix 4. Overview of all individual types of promotions (N=36) combined into three types of promotions

| **Final types of promotions** | **Combined types of promotions** | **Individual types of promotions** |
| --- | --- | --- |
| **Temporary price discount** | Discount on the purchase of 1 product | Discount on the purchase of 1 product |
| **Volume-based price promotion** | Discount on the purchase of multiple same products | Discount on the purchase of 2 same products |
|  |  | Discount on the purchase of 3 same products |
|  |  | Discount on the purchase of 4 same products |
|  |  | Discount on the purchase of 5 same products |
|  |  | Discount on the purchase of 6 same products |
|  |  | Discount on the purchase of 7 same products |
|  |  | Discount on the purchase of 8 same products |
|  |  | 2^nd^ half price |
|  |  | Other |
| **Volume-based price promotion** | Discount on the purchase of multiple different products | Discount on the purchase of 2 different products |
|  |  | Discount on the purchase of 3 different products |
|  |  | Discount on the purchase of 4 different products |
|  |  | Discount on the purchase of 5 different products |
|  |  | Discount on the purchase of 6 different products |
|  |  | Discount on the purchase of 7 different products |
|  |  | Discount on the purchase of 8 different products |
|  |  | Combination of products from different food groups |
| **Temporary price discount** | A fixed price or percentage discount | 10% discount |
|  |  | 15% discount |
|  |  | 20% discount |
|  |  | 25% discount |
|  |  | 30% discount |
|  |  | 35% discount |
|  |  | 40% discount |
|  |  | 50% discount |
|  |  | 1 euro discount |
|  |  | 2 euros discount |
|  |  | 5 euros discount |
| **Volume-based price promotion** | Buy some, get some free | Buy 1, get 1 free |
|  |  | Buy 2, get 1 free |
|  |  | Buy 2, get 2 free |
|  |  | Buy 3, get 1 free |
|  |  | Buy 4, get 2 free |
|  |  | Buy 3, get 3 free |
| **Advertised only** | No discount | No discount |
